# Beyond Critical Congenital Heart Disease: Newborn Screening Using Pulse Oximetry for Neonatal Sepsis and Respiratory Diseases in a Middle-Income Country

**DOI:** 10.1371/journal.pone.0137580

**Published:** 2015-09-11

**Authors:** Vida Jawin, Hak-Lee Ang, Asma Omar, Meow-Keong Thong

**Affiliations:** Department of Paediatrics, Faculty of Medicine, University of Malaya, Kuala Lumpur, Malaysia; NIH, UNITED STATES

## Abstract

**Background:**

Studies on pulse oximetry screening for neonatal sepsis and respiratory disease in a middle-income country are lacking. Newborn screening for critical congenital heart disease (CCHD) using pulse oximetry is an effective and life-saving strategy in developed countries. While most studies have reported false-positive results during CCHD screening, they have not elaborated on the detected disease types. We studied the effectiveness and outcomes of pulse oximetry newborn screening for non-cardiac hypoxemic diseases such as neonatal sepsis, respiratory diseases, and CCHD in a middle-income country.

**Methods and Findings:**

In a pilot study performed at the University Malaya Medical Centre (UMMC), Malaysia, all apparently healthy term newborns, delivered at UMMC were screened pre-discharge using pulse oximetry. Echocardiography was performed for newborns that had positive screening results on two separate occasions, 1-h apart. Newborns with normal echocardiograms were evaluated and treated for other non-cardiac diseases. Fifteen of 5247 term newborns had positive screening results. The median age at screening was 20 h. Thirteen newborns (0.24%) had significant non-cardiac diseases: sepsis (n = 2) and respiratory diseases (n = 11) that required hospitalization and treatment. The remaining two newborns with normal antenatal ultrasonograms had positive screening test and confirmed to have CCHD. Another 18 newborns with negative screening test were later admitted for treatment of sepsis (n = 16) and penumonia (n = 2). All newborns were treated and alive at the end of the study. The sensitivity and specificity of pulse oximetry screening for non-cardiac diseases were 42% and 99.9% respectively, and 100% and 99.7% for CCHD, respectively.

**Conclusions:**

Routine pulse oximetry screening test was effective in identifying newborns with CCHD and other hypoxemia illnesses, which may led to potential life-threatening condition. This study showed that the expanded use of pulse oximetry has immediate implications for low- and middle-income countries contemplating strategies to reduce neonatal mortality and morbidity.

**Abbreviations:**

ASD, atrial septal defect; CCHD, critical congenital heart disease; CRP, C-reactive protein; CXR, chest radiographs; NDI, neurodevelopment impairment; PPHN, persistent pulmonary hypertension of the newborn; PDA, patent ductus arteriosus; PFO, patent foramen ovale; TGA, transposition of great artery; TTN, transient tachypnoea of the newborn; VSD, ventricular septal defect.

## Introduction

Newborn screening for critical congenital heart disease (CCHD) using pulse oximetry is recognized as a highly specific, moderately sensitive, and cost-effective test that meets the criteria for universal screening. It has been endorsed in the United States and various developed countries as part of the recommended uniform screening panel for newborns [1,2]. Congenital heart disease occurs in 8–10 per 1000 live births and accounts for 3% of all infant mortalities and 46% of deaths from congenital malformations, with most deaths occurring in the first year of life [3–6]. Approximately one quarter of congenital heart disease children will have critical congenital heart disease (CCHD), which by definition requires surgery or catheter intervention in the first year of life [7,8]. Current screening methods include prenatal screening and routine newborn examination [9,10]. However, with routine fetal cardiac ultrasound during pregnancy, fewer than 50% of the CCHD cases were identified and routine newborn examinations also failed to detect 75–80% of CCHD as many newborns with CCHD have no signs that can be detected by clinical examination [11–14]. As congenital cardiac diseases and CCHD are the main cause of congenital anomalies, early detection of these non-communicable diseases will lead to improvements in neonatal mortality and morbidity in low- and middle-income countries.

Granelli et al. reported that depending on the cut-off criteria, the false positive rates of pulse oximetry screening programs vary between 0.009% and 5% with large studies reporting 0.17% and 0.3% [15,16]. Between 30% to 70% of the false positives were attributed to detection of secondary targets but details on these non-CCHD or serious non-cardiac illness were lacking or not reported as the primary objective of the studies [17,18]. Recent studies have validated the routine use of pulse oximetry in developed countries and areas of moderate altitude and the relative ease of program implementation [19–23]. However, data regarding pulse oximetry screening for CCHD, neonatal sepsis, and respiratory disorders in low- and middle-income countries is limited [24,25].

Neonatal sepsis is a common serious problem and the diagnosis may be difficult to make as the clinical manifestations are non-specific and none of the available laboratory tests could be considered as an ideal marker. However, there are evidence to link sepsis with an early onset of hypoxia. Microcirculatory dysfunction plays a pivotal role in the pathogenesis of sepsis and septic shock[26]. It is known that cytopathic hypoxia occurs in the mitochondria within cells when sepsis occurs[27]. In addition, functional shunting in the microcirculation and in the mitochondrial which lead to the deficit of oxygen extraction is also observed in sepsis and septic shock[26,28]. Therefore, pulse oximetry screening test may be beneficial in allowing an early detection of neonatal sepsis. The World Health Organization and UNICEF reported that up to three quarters of a million deaths per year worldwide are attributed to severe neonatal bacterial infections [29]. It has been estimated that in 2010 there were 6.8 million (uncertainty range: 5.4–8.1 million) cases of possible severe bacterial infection in neonates over 32 weeks gestation (or above 1,500 g), which included 1.7 million (1.1–2.4 million) cases of neonatal sepsis, 200,000 cases (21,000–350,000) of neonatal meningitis, and 510,000 cases (150,000–930,000) of neonatal pneumonia in South Asia, sub-Saharan Africa, and Latin America. In addition, moderate and severe neurodevelopment impairment (NDI) occurred in survivors of neonatal meningitis with a paucity of data on impairment for neonatal sepsis and pneumonia [30]. In selected east Asian countries, early neonatal sepsis has been reported in 4.91 per 1000 admissions with *Group B Streptococcus* as well as *Klebsiella* spp. the most common gram-negative organisms causing most deaths [31]. Infections that contributed to neonatal deaths in other developing countries ranged from 8% to 80% and accounted for as many as 42% of the deaths in the first week of life [32]. The rate of neonatal sepsis has been reported as high as 170/1000 live births (clinically diagnosed) and 5.5/1000 live births (blood culture- confirmed) [32].

In low resource settings, hand-washing routines, cleaning of the umbilical cord with chlorhexidine, clean deliveries, and improved case management using effective antibiotic treatment regimens have been identified as key steps in reducing neonatal infections [33]. To allow timely diagnosis and prevent possible NDI, pulse oximetry screening for neonatal sepsis before discharge in low- and moderate-income countries may be effective, but there are limited data [30]. One feasibility study using pulse oximetry screening for neonatal sepsis in 316 asymptomatic newborns in a low-income setting reported acceptability by mothers and healthcare professionals but cautioned that further studies were needed to assess the accuracy of the test in detecting sepsis in newborns and its clinical impact on neonatal health [34]. There are no reports of using pulse oximetry for respiratory diseases in newborns [34].

Malaysia is classified as a middle-income country with a birth rate of 500,000 per annum and an infant mortality rate of 6/1000 live births in 2009 with conditions originating in the perinatal period reported as the major cause of childhood death [35]. Currently there are no routine newborn screening programs for CCHD, neonatal sepsis, or respiratory diseases in the country. As hypoxemia is a common feature of CCHD and respiratory diseases, pulse oximetry is helpful to detect early mild cyanosis in newborns. We studied the use of pulse oximetry to screen for both CCHD and non-cardiac hypoxemic diseases such as neonatal sepsis and respiratory diseases.

## Materials and Methods

**Fig 1:**
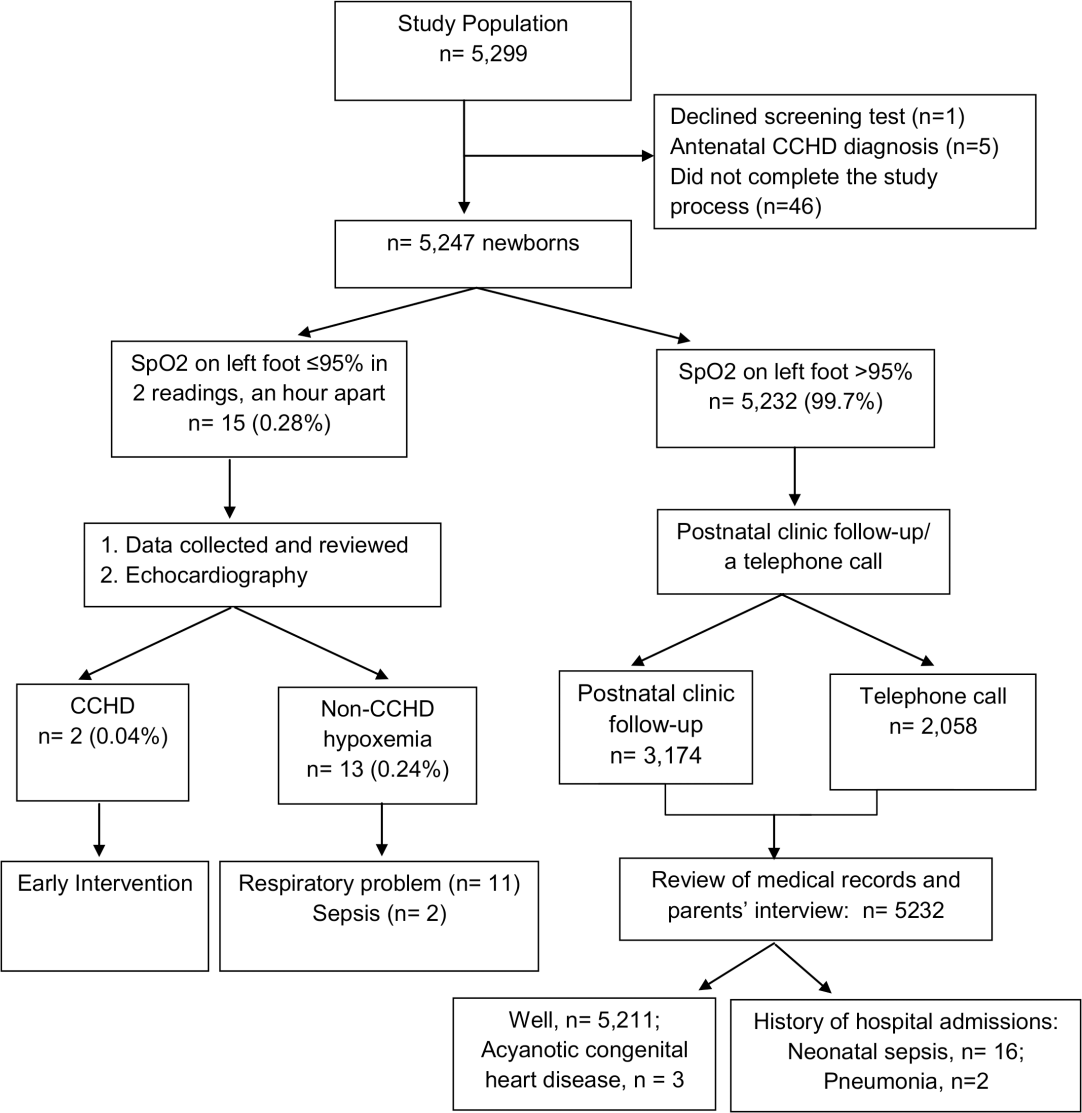
Study Protocol and Results.

All apparently healthy term newborns born at the University Malaya Medical Center (UMMC), Malaysia from September 1, 2012 to December 31, 2013, with the exception of weekends and public holidays, were enrolled prospectively in this pilot study before hospital discharge. Those newborns with an antenatal diagnosis of congenital heart disease or did not complete the study were excluded from the analysis. Institutional-review board approval was obtained from the University of Malaya Ethics Committee (Ethics Committee Reference number: 913.13) An educational brochure regarding the purpose of the study was distributed to all parents after delivery. As this screening test involved a great number of newborns, it was not feasible to obtain written consent from each legal guardian who agreed to take part in the study, hence an op-out consent applied. For those parents who agreed to participate in the study, a verbal consent was obtained on behalf of the minors and documented in the data collection sheet. For those parents of newborns who refused to participate in the screening test, an objection written consent was collected. The study was conducted according to a standardized protocol. (Fig 1). Demographics and clinical data were collected from all Infants. Infants that were screened positive were provided with immediate counselling and referred for investigation, including echocardiography within 24 h of screening.

Measurement of oxygen saturation (SpO2) was carried out within 24 h of life or before hospital discharge by a cardiology technician who had undergone an on-site supervision and training. Once competency in obtaining a reliable and accurate result using the Masimo Radical-7^TM^ SET pulse oximetry unit was achieved, the study was carried out. The M-LNCS^TM^ pulse oximetry probe was cleaned with an alcohol swab before each use to prevent cross infection and was placed on the left foot for 30 s before the reading was recorded. Newborns with an oxygen saturation reading of more than 95% were considered negative on the screening test. Those who had oxygen saturation reading less than or equal to 95% in two readings measured at least an h apart were considered to have a positive screening test and an echocardiography examination was performed by a cardiologist on the same day. In the absence of CCHD, non-cardiac hypoxemic diseases such as sepsis and respiratory diseases were identified and treated. If the repeated oxygen saturation was more than 95% on the second reading, this was also considered a negative screening test. For those newborns with a low risk test result, parents were notified and a postnatal clinic appointment was scheduled at 6 weeks of age to review the newborn’s health status. In addition, hospital admission records were reviewed to include neonates who were initially screened negative for hypoxemia but subsequently admitted for sepsis and respiratory illnesses into the analysis. During the postnatal check up, if the infants were healthy, they were discharged from the study. However, for those who did not attend the 6-week postnatal clinic follow-up, a telephone call was made to ensure their well-being and their medical records were traced.

Data analysis was performed using the statistical package for the social sciences (SPSS), version 21.0. The results and underlying causes for infants with positive tests were evaluated and studied. Sensitivity, specificity, and predictive values were calculated using standard methods for proportions.

## Results

### Study population

A total of 5,299 newborns that fulfilled the inclusion criteria were initially enrolled. Parents of one newborn declined the pulse oximetry screening test. Five newborns who were diagnosed to have CCHD antenatally were not included in the analysis. Forty-six newborns did not complete the study process at the end of 6 weeks follow-up were also excluded (Fig 1). Demographic and clinical characteristics of the remaining 5,247 newborns are described in Table 1. The median age at the time of the pulse oximetry screening test was 20 h.

**Table 1:** Demographic and clinical characteristics of newborns.

**Sex**	
Male	2556 (49%)
Female	2691 (51%)
**Race**	
Malay	3713 (70%)
Chinese	598 (12%)
Indian	512 (10%)
Others*	68 (1·3%)
Non-citizens	356 (6·7%)
**Gestational age**	
37–40 weeks (term)	4404 (83·9%)
>40 weeks (post term)	843 (16·1%)
**Birth weight**	
<2,500 gram	374 (7·1%)
2,500–4,000 gram	4791 (91·3%)
>4,000 gram	82 (1·6%)
**Mode of delivery**	
Spontaneous vaginal delivery	3482 (66·3%)
Vacuum assisted delivery	208 (3.9%)
Forceps assisted delivery	8 (0·1%)
Emergency lower section caesarean	927 (17·6%)
Elective lower section caesarean	595 (11.3%)
Born before arrival	27 (0·8%)
**Risk of sepsis** ^	
Positive	38 (0·7%)
Negative	5209 (99·3%)

Others*, Kadazan, Iban, Orang Asli; Risk of sepsis

^, rupture of membrane more than 18 hours, maternal fever, group B streptococcus on high vaginal swab.

### Newborns with pulse oximetry screening test

A total of 15 newborns (0.28%) had a positive screening test with an oxygen saturation reading ≤ 95%. Thirteen newborns (0.24%) were found to have significant problems such as sepsis (n = 2) or respiratory problems (n = 11) and there were two cases of CCHD (0.04%). (Fig 1).

The remaining 5,232 newborns had negative screening results. 3,174 newborns attended the 6 week postnatal clinic follow-up while a telephone call was made to the remaining 2058 newborns parents where their infants attended child health clinic elsewhere. 5,211 newborns were well but 18 newborns had history of post-natal hospital admission which required intervention based on medical record tracing or parents’ interview: 16 newborns fulfilled the criteria for delayed diagnosis of neonatal sepsis while another two newborns were diagnosed with congenital pneumonia and another 3 newborns were diagnosed with acyanotic congenital heart disease based on physical examination and confirmed by echocardiography. These asymptomatic patients presented with cardiac murmurs (two newborns had a ventricular septal defect (VSD) and the other had a small VSD and a patent ductus arteriosus).

Based on the cohort data of the newborns at discharge, the incidence of non-cardiac hypoxemic diseases was 2.4 per 1,000 live births. The sensitivity and specificity of pulse oximetry screening test for non cardiac hypoxemic diseases were 42% and 99.9%, respectively while the positive and negative predictive values were 86% and 99.6%, respectively. The incidence of CCHD was 0.4 in 1,000 live births. The sensitivity and specificity of the pulse oximetry screening test for CCHD were 100% and 99·7% respectively and the positive and negative predictive values were 35% and 100%, respectively.

### Non-cardiac hypoxemic disease

Our screening process detected 13 positive results due to non-cardiac diseases (Tables 2 and 3). All the newborns were clinically asymptomatic with no positive physical findings during initial examination. There were two newborns who fulfilled the diagnosis of neonatal sepsis; one newborn was confirmed to have *Streptococcus pneumoniae* septicemia with positive risk factors for sepsis, while the other had positive risk factors associated with significant rise in inflammatory markers but had negative blood culture report. Another two newborns fulfilled the criteria for congenital pneumonia with progressive tachypnoea. Chest radiographs (CXR) of one of the newborn showed pneumonia changes with a spontaneous pneumothorax of the right lung that did not require any invasive intervention; the latter had a normal chest radiograph. They were discharged after oxygen support and antibiotics therapy for 1 week.

**Table 2:** Definitions of other significant hypoxemia illnesses.

Congenital pneumonia	Raised inflammatory markers (CRP>10mg/dl)± positive culture, radiological changes on Chest X-ray, oxygen requirement (or longer than 2 h),antibiotics for ≥ 5 days
Meconium aspiration syndrome	History of meconium staining of liquor, respiratory distress, oxygen requirement (for longer than 2 h), radiological changes on Chest X-ray
Sepsis	Raised inflammatory markers (CRP>10mg/dl)± positive culture, antibiotics for ≥ 5 days
Transient tachypnoea of newborn requiring oxygen	Tachypnoea with radiological changes of fluid retention, oxygen requirement (for longer than 2 h), no rise in inflammatory markers or positive culture
Persistent pulmonary hypertension of newborn	Preductal and postductal difference in saturations with echocardiogram finding of significant tricuspid regurgitation and evidence of right to left shunt across the PFO and or PDA

CRP, c-reactive protein; PFO, patent oramen ovale; PDA, patent ductus arteriosus. *Table adapted from source file of*: *Anju Singh*, *Shree Vishna Rasiah*, *Andrew K Ewer*. *The impact of routine predischarge pulse oximetry screening in a regional neonatal unit*. *Arch Dis Child Fetal Neonatal Ed*.*2014 Jul; 99(4)*: *F297-302*

**Table 3:** Non-Cardiac Hypoxemia Diseases.

No	Diagnosis	Risk factor	PO1 foot, PO2 foot	Age of screening (hours of life)	Routine physical examination	Echocardiography	Investigation	Treatment	Outcome
1	Neonatal sepsis	Risk factor of sepsis +	86%, 84%	16	Normal	Small PFO/PDA	Streptococcus pneumonia (blood culture)	Antibiotics	Alive
2	Neonatal sepsis	Risk factor of sepsis +	92%, 93%	21	Normal	Small muscular VSD	Sterile blood culture	Antibiotics	Alive
3	Down syndrome with primary PPHN	Nil	87%, 88%	6	Normal	High Tricuspid regurgitation jet 60mmHg, large PDA 5.7mm	CXR: normal Sterile blood culture	Ventilated Inhaled nitric oxide Inotropic support Antibiotics	Alive
4	PPHN with polycythemia	Nil	87%, 81%	8	Normal	Small PFO/PDA	Hematocrite: 0·8	Partial exchange at 10 hours of life	Alive
5	TTN	Nil	93%, 95%	22	Normal	Small PFO/PDA	CXR: normal	Oxygen support	Alive
6	TTN	Nil	82%, 83%	20	Normal	Small PFO/PDA	CXR: normal	Oxygen support	Alive
7	TTN	Nil	89%, 86%	24	Normal	Small apical VSD, small PDA	CXR: normal	Oxygen support	Alive
8	TTN	Nil	87%, 90%	25	Normal	Small PFO/PDA	CXR: normal	Oxygen support	Alive
9	Congenital pneumonia	Risk factor of sepsis +	86%, 86%	14	Normal	Mid muscular VSD 1.5mm	CXR: small spontaneous pneumothorax right lung	Oxygen support Antibiotics	Alive
10	Congenital pneumonia	Risk factor of sepsis +	82%, 85%	7	Normal	Small apical VSD	CXR: bilateral diffuse patchy opacities	Oxygen support Antibiotics	Alive
11	Meconium aspiration syndrome	Light meconium stained liquor delivery	92%, 93%	5	Normal	Small PFO/PDA.	CXR: bilateral diffuse patchy opacities	Oxygen support Antibiotics	Alive
12	Meconium aspiration syndrome	meconium stained liquor delivery	84%, 86%	5	Normal	Atrial septal defect (ASD) 4 mm, PDA 3 mm. Tricuspid regurgitation jet 30mmHg	CXR: bilateral diffuse patchy opacities	Ventilated at 12 hours of life Antibiotics	Alive
13	Hypoplastic right lung with VACTERL association	Nil	92%, 94%	13	Right forearm deformity	Mesocardia, small PDA	Computerized tomograms of thorax: right lung hypoplasia. Skeletal survey: absent right radius, T6 hemivetebra Ultrasound of kidney: bilateral horseshoe kidneys	Continuous bilevel positive airway pressure (BIPAP)	Alive

PO1, first pulse oximetry reading; PO2, second pulse oximetry reading; SpO2, oxygen saturation; PFO, patent foramen ovale; PDA, patent ductus arteriosus; CXR, chest x-ray; VSD, ventricular septal defect; PPHN, persistent pulmonary hypertension of newborn; TTN, transient tacypnoea of newborn; VACTERL, vertebral anomalies, anal atresia, cardia defects, tracheoesophageal fistula and or esophageal atresia, renal and radial anomalies, and limb defects

The other two newborns were diagnosed with persistent pulmonary hypertension of the newborn (PPHN), a clinical condition in which pre- and post-ductal oxygen saturation differences are ≥10%. One of the newborn with Down syndrome was diagnosed with primary PPHN, which required invasive ventilation and inhaled nitric oxide therapy. The patient was subsequently discharged at one month of age. Another newborn had PPHN secondary to polycythemia and a partial exchange transfusion was required at 12 h of age. There were two apparently healthy newborns with a history of meconium stained liquid during delivery–and later diagnosed with meconium aspiration syndrome. Those newborns had developed respiratory distress who required oxygen treatment and their chest radiograph showed the radiological changes. There was no PPHN in these patients and both newborns were healthy and discharged at 1 week of age.

An interesting case was a newborn with a deformity of the right forearm was screened positive. The patient was initially well but later developed respiratory distress. Investigations performed, including a computerized tomogram (CT) of the thorax, showed right lung hypoplasia. Other findings included a sixth thoracic hemivertebrae and bilateral horseshoe kidneys. A clinical diagnosis of VACTERL association (vertebral anomalies, anal atresia, cardia defects, tracheoesophageal fistula and or esophageal atresia, renal and radial anomalies, and limb defects) was made. The patient required noninvasive ventilatory support and was discharged at 1 month of age with long-term home bilevel positive airway pressure support.

Four newborns were treated as transient tachypnoea of newborn (TTN) which required oxygen therapy for 1–2 days. Chest X-rays were normal and there was no positive culture or rise in inflammatory markers. The TTN resolved spontaneously after 48–72 hours.

Four of 13 newborns with other hypoxemia illensses were found to have incidental VSD (n = 2 apical VSD) and mid muscular VSD (n = 2 muscular VSD) on echocardiography, which were non-critical congenital heart diseases.The echocardiography of the other newborns showed a small patent foramen ovale (PFO) and patent ductus arteriosus (PDA) which were asymptomatic and considered normal findings in newborns within the first 24 h of life. A repeat echocardiography at 6 weeks old showed a complete resolution of the defect.

### CCHD diagnoses

Two newborns who had normal antenatal and fetal cardiac ultrasonograms were screened positive using pulse oximetry and later confirmed to have CCHD on postnatal echocardiogram. One patient had pulmonary atresia with VSD and another had pulmonary atresia, transposition of the great artery (TGA) and double outlet right ventricle. The initial routine postnatal newborn examinations in these newborns were normal and cyanosis was not detected. After given an appropriate treatment, all newborns with CCHD were alive at the conclusion of this study. (Table 4).

**Table 4:** Newborns with CCHD.

No	CCHD lesions (echocardiography)	Antenatal ultrasonograms	Age of screening (hours of life)	PO1 foot, PO2 foot	SpO2 at 24 hours of life	Routine physical examination	Treatment	Outcome
1	Pulmonary atresia with VSD/ASD	Normal	9	65%, 74%	87–91%	Normal	Prostaglandin E2 at 14 hours of life. PDA stenting at 1 week of life	Alive
2	Double outlet right ventricle/Pulmonary atresia/TGA VSD/ASD/ PDA	Normal	6	83%, 86%	89–92%	Normal	Prostaglandin E2 at 9 hours of life, Blalock-Taussig shunt at 3 months old.	Alive

PO1, first pulse oximetry reading; PO2, second pulse oximetry reading; SpO2, oxygen saturation; VSD, ventricular septal defect; ASD, atrial septal defect; TGA, transposition of great artery; PDA, patent ductus arteriosus

## Discussion

The majority of studies on newborn screening using pulse oximetry have been focused on the detection of CCHD. However, little is known about non-cardiac hypoxemia illnesses, which are the secondary benefits of pulse oximetry screening test. In this study, we evaluated the outcome of newborn screening for various non-cardiac hypoxemia diseases using pulse oximetry and showed that this approach has the potential to reduce neonatal infant mortality and morbidity resulting from neonatal sepsis and various respiratory diseases of the newborn.

Pulse oximetry screening test has detected a number of non-cardiac diseases with hypoxemia (0.25%) such as neonatal sepsis and respiratory problems which were asymptomatic on physical examination but required immediate medical attention and treatment. As respiratory diseases and infections contribute to the highest mortality rate during the neonatal period in low resources and middle-income countries, pulse oximetry has an additional advantage to detect these non-CCHD and serious conditions earlier before hospital discharge, so that appropriate treatment can be instituted. Whilst some authorities may consider transient tachypnea of newborns (TTN) and mild meconium aspiration syndrome (MAS) as non-life threatening, it is unclear whether early hypoxemic state due to TTN and mild MAS or tbeirlate diagnosis may contribute to long-term morbidity, such as neurologic damage and developmental disabilities. Hence, further studies are required to investigate the effect of persistent mild newborn hypoxemia.

As perinatal complications and newborn infection rates are high in developing countries, pulse oximetry screening for CCHD may be used concurrently to screen for other newborn non-cardiac hypoxemic conditions. This may help low resource countries to reduce infant mortality and morbidity rates and to achieve the Millennium Development Goal 4, target 4A. This has been demonstrated by a feasibility study in Tanzania with 316 newborns, which detected eight newborns with sepsis, including four were detected before 12 h of life [6]. With the additional advantage of detecting these non-cardiac neonatal diseases, the percentage of false positive pulse oximetry screening results will be reduced.

In this study, the true positive for non-CCHD hypoxemia illnesses and CCHD were 13 and 2 neonates, respectively with no false positive result. Out of 5247, there were 18 false negative results (0.34%) which included 2 and 16 neonates admitted and treated for pneumonia and neonatal sepsis, respectively. Be that as it may, pulse oximetry successfully detected early 13 out of 31 (42%) neonates with life-threatening conditions. The earlier diagnosis and treatment of respiratory illnesses and neonatal sepsis reduced morbidity and further studies will be required to ascertain the long term outcome of this cohort of patients. In addition, while there was no mortality reported resulting from respiratory diseases and neonatal sepsis, it is possible that in a larger study involving other low or moderate resource countries, infant mortality rates may be reduced.

Although there was sufficient evidence for CCHD screening using pulse oximetry, this pilot study indicated an even greater potential advantage of using pulse oximetry to screen for neonatal sepsis and respiratory diseases. On a global scale, as these diseases account for a large number of neonatal mortality and morbidity particularly in low resource settings, there is a potential for huge gains using pulse oximetry screening for respiratory diseases and neonatal sepsis.

Parental acceptance of newborn screening in our setting was high as only the parents of one newborn chose to opt out from the screening program. The study was supported by health professionals, and the test was highly accurate. The total amount of time spent for the training of the cardiology technician for the study was four hours. This included 2 hours for explanation and familiarisation of the protocol and demonstration of the correct technique of measuring pulse oximetry readings. This was followed by on-site supervision to ascertain the competency of the technician in obtaining a reliable and consistent result. On the average, the time required for pulse oximetry screening test for each newborn was 5 minutes. As the number of positive screening case detected was 15 cases over 16 months, the number of screening tests which require further assessment and observation was <1 case/month. This did not require additional manpower and the time consumed was negligible. On the basis of our finding, pulse oximetry screening will require no or minimal additional resource in the hospital that had paediatric cardiologist services. However further study will be required to ascertain the resources needed in the hospital without paediatric cardiologist service. We concluded that pulse oximetry screening test was simple, feasible and did not appear to overload clinic services. While antenatal or fetal cardiac echocardiography may detect those with gross four chamber anomalies, there is a strong likelihood of missing those with outflow tract defects. In addition, other confounding factors leading to false negative antenatal ultrasound findings were the dependence on the level of expertise of the ultrasonographer, gestational age of the fetus, fetal position, and the type of heart defect. The majority of the newborns with CCHD remained asymptomatic after birth due to the presence of persistent fetal transitional circulation. The presence of cyanosis may not be apparently visible in newborns with darker skin complexion. Therefore, these newborns would likely be discharged with undetected CCHD if pulse oximetry was not performed.

This study also showed the importance of post screening follow-up as well as a need to establish a registry for CCHD and non-CCHD hypoxemic diseases to further evaluate the cost-effectiveness of this screening strategy before a nation-wide or national program is launched. Detailed analyses of the types of non-cardiac hypoxemic diseases detected may help in the appropriate allocation of resources to neonatal care.

We confirmed that the pulse oximetry screening test for newborns had a significant impact on neonatal health. The test was highly accurate and acceptable to both parents and health professionals. This test is able to confer the additional benefit to detect non-cardiac hypoxemic diseases such as neonatal sepsis and respiratory diseases, especially in low- and middle-income countries. A similar larger multicenter international study in low resource or developing countries should be done to confirm the above observations as well as to determine the cost-effectiveness of this strategy.

## Supporting Information

S1 Table List of positive pulse oximetry screening result. (DOCX)

S2 Table List of false negative pulse oximetry screening result. (DOCX)

## References

[1] Thangaratinam S, Brown K, Zamora J, Khan KS, Ewer AK. Pulse oximetry screening for critical congenital heart defects in asymptomatic newborn babies: a systematic review and meta-analysis. Lancet. 2012; 379(9835):2459–64. 10.1016/S0140-6736(12)60107-X 22554860

[2] Sebelius K. HHS Secretary adopts recommendation to add Critical Congenital Heart Disease to the Recommended Uniform Screening Panel 9 21, 2011 Washington, DC: US Department of Health and Human Services 2011. Available: http://www.hrsa.gov/advisorycommittees/mchbadvisory/heritabledisorders/recommendations/correspondence/cyanoticheartsecre09212011.pdf.

[3] Payne RM, Johnson MC, Grant JW, Strauss AW. Toward a molecular understanding of congenital heart disease. Circulation 1995; 91(2): 494–504. 7805255 10.1161/01.cir.91.2.494

[4] Ainsworth SB, Wyllie JP, Wren C. Prevalence and clinical significance of cardiac murmurs in neonates. Arch Dis Child Fetal Neonatal 1999; 80(1): 43–45. 10.1136/fn.80.1.f43 PMC1720873 10325811

[5] Wren C, Richmond S, Donaldson L. Presentation of congenital heart disease in Infancy: implications for routine examination. Arch Dis Child Fetal Neonatal 1999; 80(1b): 49–53. 10.1136/fn.80.1.f49 PMC1720871 10325813

[6] Knowles R, Griebsch I, Dezateux C, Brown J, Bull C, Wren C. Newborn screening for congenital heart defects: a systematic review and cost-effectveness analysis. Health Technol Assess 2005; 9(44): 1–152. 10.3310/hta9440 16297355

[7] Talner CN. Report of the New England Infant Cardiac Program, by Donald C. Flyer MD. Pediatrics 1980; 65(suppl): 375–461. *Pediatrics* 1998; 102(pt2): 258–259. 9651450

[8] Schultz AH, Localio AR, Clark BJ, Ravishankar C, Videon N, Kimmel SE. Epidemiologic features of the presentation of critical congenital heart disease: implications for screening. Pediatrics 2008; 121(4): 751–757. 18381540 10.1542/peds.2007-0421

[9] Mahle WT, Newburger JW, Matherne GP, Smith FC, Hoke TR, Koppel R, et al Role of pulse oximetry in examining newborns for congenital heart disease: a scientific statement from the American Heart Association and American Academy of Pediatrics. Circulation 2009; 120: 447–58. 19581492 10.1161/CIRCULATIONAHA.109.192576

[10] Wren C, Richmond S, Donaldson L. Presentation of congenital heart disease in infancy: implications for routine examination. Arch Dis Child Fetal Neonatal Ed. 1999; 80(1): F49–F53. 10325813 10.1136/fn.80.1.f49 PMC1720871

[11] Singh A, Rasiah SV, Ewer AK. The impact of routine predischarge pulse oximetry screening in a regional neonatal unit. Arch Disc Child Fetal Neonatal Ed. 2014 7; 99(4):F297–302. Epub 2014 Mar 19. 10.1136/archdischild-2013-305657 24646619

[12] Ewer AK, Furmston AT, Middleton LJ, Deeks JJ, Daniels JP, Pattison HM, et al Pulse oximetry as a screening test for congenital heart defects in newborn infants: a test accuracy study with evaluation of acceptability and cost-effectiveness. Health Technol Assess. 2012;16(2):v–xiii, 1–184. 10.3310/hta16020 22284744

[13] Hunter S, Heads A, Wyllie J, Robson S. Prenatal diagnosis of congenital heart disease in the northern region of England: benefits of a training programme for obstetric ultrasonographers. Heart 2000; 84(3): 294–298. 10956294 10.1136/heart.84.3.294 PMC1760944

[14] Klein SK, Cans C, Robert E, Jouk PS. Efficacy of routine fetal ultrasound screening for congenital heart disease in Isère County, France. Prenat Diagn 1999; 19(4): 318–322. 10327135 10.1002/(sici)1097-0223(199904)19:4<318::aid-pd538>3.0.co;2-x

[15] Ewer AK. Pulse oximetry screening for critical congenital heart defects.Should it be routine? Arch Dis Child Fetal Neonatal Ed 2014; 99: 93–95. 10.1136/archdischild-2013-303968 23934364

[16] Ewer AK. Pulse oximetry screening: do we have enough evidence now? Lancet 2014 8 30; 384(9945):725–6. 10.1016/S0140-6736(14)60575-4 Epub 2014 Apr 22 24768154

[17] Randall P, Brealey S, Hahn S, Khan KS, Parsons JM. Accuracy of fetal echocardiography in the routine detection of congenital heart disease among unselected and low risk populations: a systematic review. BJOG 2005; 112(2): 24–30 15663393 10.1111/j.1471-0528.2004.00295.x

[18] Riede FT, Wörner C, Dähnert I, Mockel A, Kostelka M, Schneider P. Effectiveness of neonatal pulse oximetry screening for detection of critical congenital heart disease in daily clinical routine-results from a prospective multicenter study. Eur J Pediatr. 2010; 169(8): 975–981. 20195633 10.1007/s00431-010-1160-4 PMC2890074

[19] Ewer AK. Review of pulse oximetry screening for critical congenital heart disease in newborn infant. Arch Dis Child Fetal Neonatal Ed 2014; 99: F93–F95. 23934364 10.1136/archdischild-2013-303968

[20] Wright J. Kohn M, Niermeyer S, Rausch C. Feasibility of critical congenital heart disease newborn screening at moderate altitude. Pediatrics 2014; 133: 3 e561–e569. 10.1542/peds.2013-3284 24567022

[21] Andrews JP, Ross AS, Salazar MA, Tracy NA, Burke BR Jr. Smooth implementation of critical congenital heart defect screening in a newborn nursery. Clin Pediatr 2014; 53(2): 173–176. 10.1177/0009922813502850 24037922

[22] Ruangritmachai C, Bunjapamai W, Pongpanich B. Pulse oximetry screening for clinically unzecognized congenital heart disease in the newborns. Pediatr Cardiol 2007; 9(1): 10–15. PMC3232575 22368668

[23] Zhao Q.M., Ma X.J., Ge X.L., Liu F, Yan WL, Wu L, et al the Neonatal Congenital Heart Disease screening group. Pulse oximetry with clinical assessment to screen for congenital heart disease in neonates in China: a prospective study. Lancet 2014; 384: 747–754. 24768155 10.1016/S0140-6736(14)60198-7

[24] de-Wahl Granelli A, Wennergren M, Sandberg K, Mellander M, Bejlum C, Inganas L, et al Impact of pulse oximetry screening on the detection of duct dependent congenital heart disease: a Swedish prospective screening study in 39,821 newborns.BMJ 2009; 338: a3037. 19131383 10.1136/bmj.a3037 PMC2627280

[25] Ewer AK. Pulse oximetry screening for critical congenital heart defects.Should it be routine? Arch Dis Child Fetal Neonatal Ed 2014; 99: 93–95. 10.1136/archdischild-2013-303968 23934364

[26] Koh IH, Menchaca-Diaz JL, Koh TH, Souza RL, Shu CM, Rogerio VE, et al Microcirculatory evaluation in sepsis: a difficult task. Shock. 2010; 34 Suppl 1:27–33. 10.1097/SHK.0b013e3181e7e80c 20523273

[27] Fink M. Cytopathic hypoxia in sepsis. Acta Anaesthesiol Suppl. 1997; 110:87–95. 10.1111/j.1399-6576.1997.tb05514.x 9248546

[28] Ince C, Mik EG. Microcirculatory and mithochondrial hypoxia in sepsis, shock and resuscitation. J Appl Physiol (1985) 2015. 10.1152/japplphysiol.00298.2015 26066826

[29] Liu L, Johnson HL, Cousens S, Perin J, Scott S, lawn JE, et al; Child Health Epidemiology eference Group of WHO and UNICEF. Global, regional, and national causes of child mortality: an updated systematic analysis for 2010 with time trends since 2000. Lancet 2012; 379:2151–61. 22579125 10.1016/S0140-6736(12)60560-1

[30] Seale AC, Blencowe H, Zaidi A, Ganatra H, Syed S, Engmann C, et al and on behalf of the neonatal infections estimation team. Neonatal severe bacterial infection impairment estimates in South Asia, sub-Saharan Africa, and Latin America for 2010. Pediatr Res 2013; 74: 73–85 24366464 10.1038/pr.2013.207 PMC3873707

[31] Al-Taiar A, Hammoud MS, Cuiqing L, Lee JK, Lui KM, Nakwan N, et al Neonatal infections in China, Malaysia, Hong Kong and Thailand. Arch Dis Child Fetal Neonatal Ed 2013; 98: F249–F255. 22942104 10.1136/archdischild-2012-301767

[32] Thaver Durrane, Zaidi Anita. Burdens of neonatal infections in developing countries: A review of evidence from community-based studies. Pediatr Infectious Disease J. 2009; 28: pp S3–S9. 19106760 10.1097/INF.0b013e3181958755

[33] Seale AC, Berkley JA. Managing severe infection in infancy in resource poor settings. Early Hum Dev 2012; 88: 957–60. 23031387 10.1016/j.earlhumdev.2012.09.005 PMC3507620

[34] King EM, Lieu C, Kasasa A, Ewer AK, Thangaratinam S. Pulse oximetry as a screening tool to detect hypoxia associated with early-onset sepsis in asymptomatic newborns: A feasibility study in a low-income country. British Journal of Medicine & Medical Research 2014; 4(5):1115–28.

[35] Level & Trends in Child Mortality; Estimates Developed by the UN Inter-agency Group for Child Mortality Estimation. 2010.

