# Supplementary material for: Beyond Critical Congenital Heart Disease: Newborn Screening Using Pulse Oximetry for Neonatal Sepsis and Respiratory Diseases in a Middle-Income Country
Source: PLoS One. 2015 Sep 11;10(9):e0137580. doi: 10.1371/journal.pone.0137580 (PMC4567069; doi:10.1371/journal.pone.0137580)
Supplement: S1 Table — (DOCX) [file pone.0137580.s001.docx]

S1 Table. List of positive pulse oximetry screening result

| Number | Hour of Life | SPO2 Foot (1) | SPO2 Foot (2) | Echocardiography | Final Diagnosis | Outcome |
| --- | --- | --- | --- | --- | --- | --- |
| 1 | 17 | 86 | 84 | small PFO, PDA 1.5mm with bidirectional shunt. | Neonatal sepsis | Alive |
| 2 | 19 | 87 | 88 | PFO 2 mm, PDA 3mm | PPHN | Alive |
| 3 | 20 | 82 | 83 | small PFO 2.6mm, Tiny PDA with bidirectional shunt. | TTN | Alive |
| 4 | 14 | 86 | 86 | PFO 3.5mm,mid muscular VSD 1.5mm. | Congenital pneumonia | Alive |
| 5 | 15 | 87 | 81 | PFO , PDA 1.5mm. | PPHN | Alive |
| 6 | 17 | 82 | 85 | small PFO 2mm, small apical muscular VSD 1mm | Congenital pneumonia | Alive |
| 7 | 24 | 87 | 90 | PFO 2mm, PDA 1.5mm | TTN | Alive |
| 8 | 20 | 84 | 86 | moderate ASD 6.5mm ,PDA 3mm | MAS | Alive |
| 9 | 13 | 92 | 93 | PFO 2mm, PDA 1mm | MAS | Alive |
| 10 | 26 | 89 | 86 | tiny PDA, PFO 3mm. | TTN | Alive |
| 11 | 22 | 92 | 93 | tiny PFO, tiny muscular VSD | Neonatal sepsis | Alive |
| 12 | 21 | 93 | 95 | PDA 2.3mm/PFO 3mm | TTN | Alive |
| 13 | 18 | 92 | 94 | PDA 2.6mm | VACTERL association | Alive |
| 14 | 18 | 65 | 74 | small PFO 3mm, large outlet VSD 7mm with overiding aorta, pulmonary atresia(absent main pulm artery), right PA/left PA : 3.7/3.7mm, tortuous PDA, left sided aortic arch, no coarctation of aorta | PA with ASD/VSD | Alive |
| 15 | 19 | 83 | 86 | DORV,TGA,PA,ASD (large almost single atrium), VSD, tortuous PDA. | DORV/PA/TGA/VSD/ ASD/PDA | Alive |

PO1, first pulse oximetry reading; PO2, second pulse oximetry reading; SpO2, oxygen saturation; PFO, patent foramen ovale; PDA, patent ductus arteriosus; CXR, chest x-ray; VSD, ventricular septal defect; PPHN, persistent pulmonary hypertension of newborn; TTN, transient tacypnoea of newborn; VACTERL, vertebral anomalies, anal atresia, cardia defects, tracheoesophageal fistula and or esophageal atresia, renal and radial anomalies, and limb defects; ASD, atrial septal defect; TGA, transposition of great artery.
