# Supplementary material for: Beyond Critical Congenital Heart Disease: Newborn Screening Using Pulse Oximetry for Neonatal Sepsis and Respiratory Diseases in a Middle-Income Country
Source: PLoS One. 2015 Sep 11;10(9):e0137580. doi: 10.1371/journal.pone.0137580 (PMC4567069; doi:10.1371/journal.pone.0137580)
Supplement: S2 Table — (DOCX) [file pone.0137580.s002.docx]

S2 Table. List of false negative pulse oximetry screening result

| Number | Hour of Life | SPO2 Foot (1) | Review of medical record/ parents' interview | Outcome |
| --- | --- | --- | --- | --- |
| 1 | 20 | 99 | History of re-admission for Neonatal sepsis | Alive |
| 2 | 2 | 100 | History of re-admission for Neonatal sepsis | Alive |
| 3 | 4 | 99 | History of re-admission for Neonatal sepsis | Alive |
| 4 | 15 | 100 | History of re-admission for Neonatal sepsis | Alive |
| 5 | 17 | 98 | History of re-admission for Neonatal sepsis | Alive |
| 6 | 18 | 99 | History of re-admission for Neonatal sepsis | Alive |
| 7 | 22 | 99 | History of re-admission for Neonatal sepsis | Alive |
| 8 | 24 | 99 | History of re-admission for Neonatal sepsis | Alive |
| 9 | 13 | 98 | History of re-admission for Neonatal sepsis | Alive |
| 10 | 15 | 99 | History of re-admission for Neonatal sepsis | Alive |
| 11 | 17 | 99 | History of re-admission for Neonatal sepsis | Alive |
| 12 | 15 | 100 | History of re-admission for Neonatal sepsis | Alive |
| 13 | 22 | 100 | History of re-admission for Neonatal sepsis | Alive |
| 14 | 20 | 100 | History of re-admission for Neonatal sepsis | Alive |
| 15 | 26 | 100 | History of re-admission for Neonatal sepsis | Alive |
| 16 | 22 | 99 | History of re-admission for Neonatal sepsis | Alive |
| 17 | 20 | 99 | History of re-admission for congenital pneumonia | Alive |
| 18 | 17 | 99 | History of re-admission for congenital pneumonia | Alive |
